# The Efficacy of Denosumab in Treating Spinal Aneurysmal Bone Cyst: A Case Report

**DOI:** 10.7759/cureus.39954

**Published:** 2023-06-04

**Authors:** Shinji Kotaka, Yasushi Fujiwara, Ryo Ota, Hideki Manabe, Nobuo Adachi

**Affiliations:** 1 Orthopaedic and Microscopic Spine and Spinal Cord Surgery Center, Hiroshima City North Medical Center Asa Citizens Hospital, Hiroshima, JPN; 2 Department of Orthopaedic Surgery, Hiroshima Hiramatsu Hospital, Hiroshima, JPN; 3 Department of Orthopaedic Surgery, Graduate School of Biomedical and Health Sciences, Hiroshima University, Hiroshima, JPN

**Keywords:** chemotherapy, spine, non-operative treatment, denosumab, aneurysmal bone cysts

## Abstract

The optimal treatment for aneurysmal bone cysts (ABCs) of the spine remains controversial. No treatment guidelines exist for the use of denosumab in aneurysmal bone cysts. In this report, we describe the results from a representative case and compare our experience with those of previously published reports.

A 38-year-old male was referred for pain in the lower back and left leg. Radiographs and a needle biopsy specimen revealed a lumbar aneurysmal bone cyst, which was treated with denosumab chemotherapy. The pain in the lower back and left leg gradually improved, and at 16 weeks, the symptoms had resolved. Once a satisfactory local effect was achieved, denosumab therapy was discontinued. However, the erosive lesion subsequently expanded. After re-initiation of treatment, there was no subsequent evidence of recurrence.

Single-therapy denosumab is an option for aneurysmal bone cysts. However, recurrences have been documented after denosumab termination, and the timing for cessation of denosumab is controversial.

## Introduction

Aneurysmal bone cysts (ABCs) are rare, benign skeletal tumors. These tumors account for 1%-2% of all primary bone tumors, usually present in the first two decades of life, exhibit a slight female preponderance, and occur in the vertebral column in approximately 3%-30% of cases. Although benign, the lesions may involve all parts of the vertebrae, including the vertebral body, and frequently the posterior elements [1]. This can result in large lesions affecting the spine, leading to significant morbidity.

There are various therapeutic options for ABCs; however, the optimal treatment for ABCs of the spine remains controversial. Treatments include curettage, excision, embolization, radiotherapy, and chemotherapy. The decision regarding the optimal option is based on personal and institutional experience and preferences, the location of the mass, and lesion size in most cases [2]. According to recent reports, intralesional curettage or surgical resection is still the predominantly performed therapy; however, the recurrence rates are high, and it is reported that “en bloc” resection is required for preventing recurrence [3]. In addition, some reports suggest that radiotherapy, chemotherapy, and embolization as adjuvant therapies reduce the recurrence rate after surgery [4,5]. However, these could lead to high surgical morbidity for patients. Furthermore, it is sometimes difficult to perform surgeries in the spine, as the tumor could affect the nervous tissues, resulting in postoperative neurological deficits. There are some reports of various non-surgical treatment modalities comprising sclerotherapy, radionuclide ablation, and embolization for ABC of the spine [6]. The optimal therapy is chosen after carefully considering the patient’s age and the location, size, vascularity, and degree of invasion of the lesions.

Denosumab has recently been used to treat ABCs [5,7]. Denosumab has been approved for various disorders including postmenopausal osteoporosis, cancer-related bone injuries, and giant cell tumor [8]. No treatment guidelines exist for the use of denosumab in ABC. However, a case series of patients with ABCs treated with denosumab has previously been published. Ghermandi et al. [9] reported two cases of vertebral ABC that were unresponsive to selective arterial embolization and were treated with denosumab; Dubory et al. [5] reported two patients with ABC that underwent treatment with denosumab as neoadjuvant therapy. Both reports indicated a favorable response.

The aim of this study was to report the results from a representative case and compare our experience with those of previously published reports.

## Case presentation

A 38-year-old Japanese male presented with gradually progressive pain in the lower back and left leg for two months, and he was referred to our hospital for management. He had no other medical history. Examination of the spine showed localized tenderness of the L3 spinous process without a palpable mass. He had no lower limb muscle weakness or sensory disturbances. The Japanese Orthopedic Association score was 10/17.

**Figure 1:**
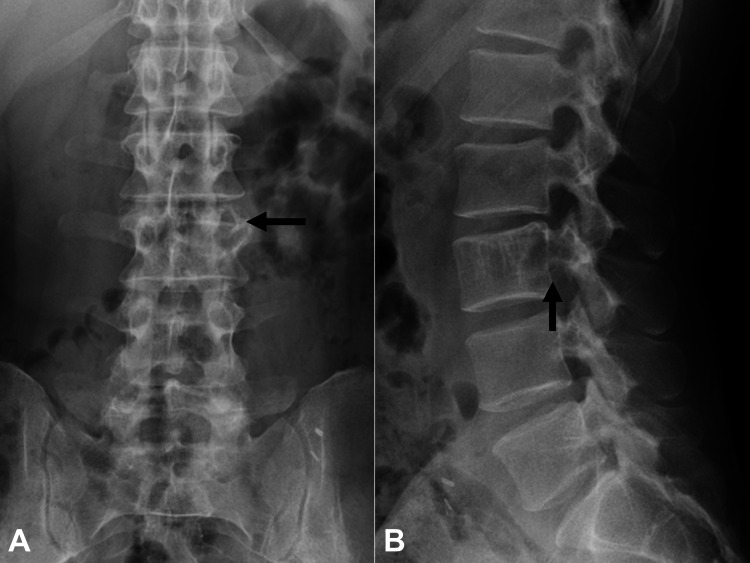
Plain lumbar radiographs of the lumbar spine. Osteolytic lesion involving the left L3 posterior portion of the vertebra with a pedicle sign (arrows). (A) Anterior-posterior view. (B) Medial-lateral view.

Plain lumbar radiographs showed an osteolytic lesion involving the left posterior portion of the L3 vertebra with a pedicle sign observed in the anteroposterior view (Figure 1).

**Figure 2:**
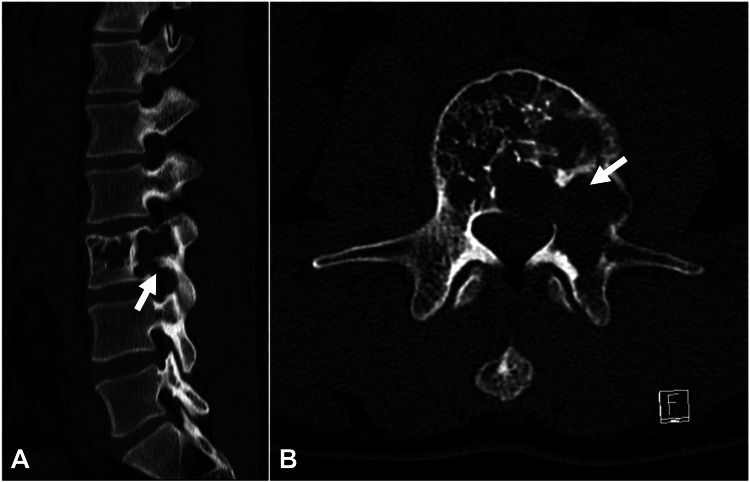
Reconstructed computed tomography. Expansile and destructive bone lesion at the vertebral body and left pedicle of the L3 (arrows). (A) Sagittal view. (B) Axial view.

Reconstructed computed tomography (CT) images revealed an expansile and destructive bone lesion in the body and left pedicle of the L3 vertebra (Figure 2).

**Figure 3:**
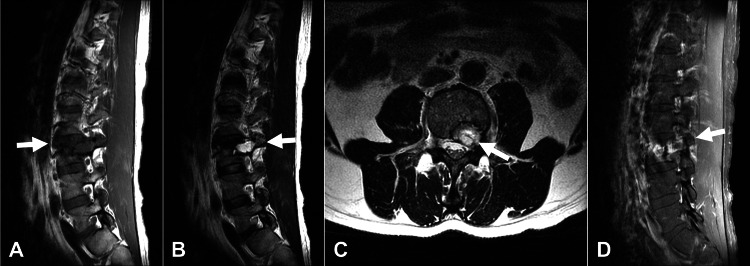
Magnetic resonance imaging of the lumbar spine. The anterior portion of the L3 vertebra with iso- and high-signal intensities on (A) T1-weighted image and (B) T2-weighted image, respectively (arrows). (B) Fluid-fluid level in the posterior portion of the vertebral body and left pedicle (arrow). (C) The lesion compresses to the left side of the dura and left L4 nerve root at the lateral recess (arrow). (D) The anterior portion of the vertebral body contrasted on gadolinium-enhanced MRI (arrows). MRI: magnetic resonance imaging

Sagittal magnetic resonance imaging showed iso- and high-signal intensities on T1- and T2-weighted images, respectively, in the anterior portion of the L3 vertebra (Figure 3A (T1) and Figure 3B (T2)). A fluid-fluid level was noted in the posterior portion of the vertebral body and left pedicle (Figure 3B (arrow)). The lesion compressed the left side of the dura and L4 nerve root in the lateral recess (Figure 3C (arrow)). Furthermore, contrast enhancement was clearly observed in the anterior portion of the vertebral body in gadolinium-enhanced images (Figure 3D).

A needle biopsy was performed under CT fluoroscopy, and histological reports confirmed the diagnosis of an ABC without malignant features.

Surgery was planned to prevent neurological symptoms and pathological fractures. However, tumor removal was difficult because of its proximity to the spinal canal and foramina, and due to his fear of surgical intervention, he wished for the treatment of denosumab based on sufficient informed consent. Therefore, the patient was treated with denosumab chemotherapy. The protocol used in treating giant cell tumors was followed: 120 mg denosumab was administered subcutaneously on days 1, 8, 15, and 29; subsequently, 60 mg denosumab was administered subcutaneously once every four weeks; and calcium carbonate (600 mg) and natural vitamin D (400 IU) were dispensed orally once daily to prevent hypocalcemia during denosumab administration.

**Figure 4:**
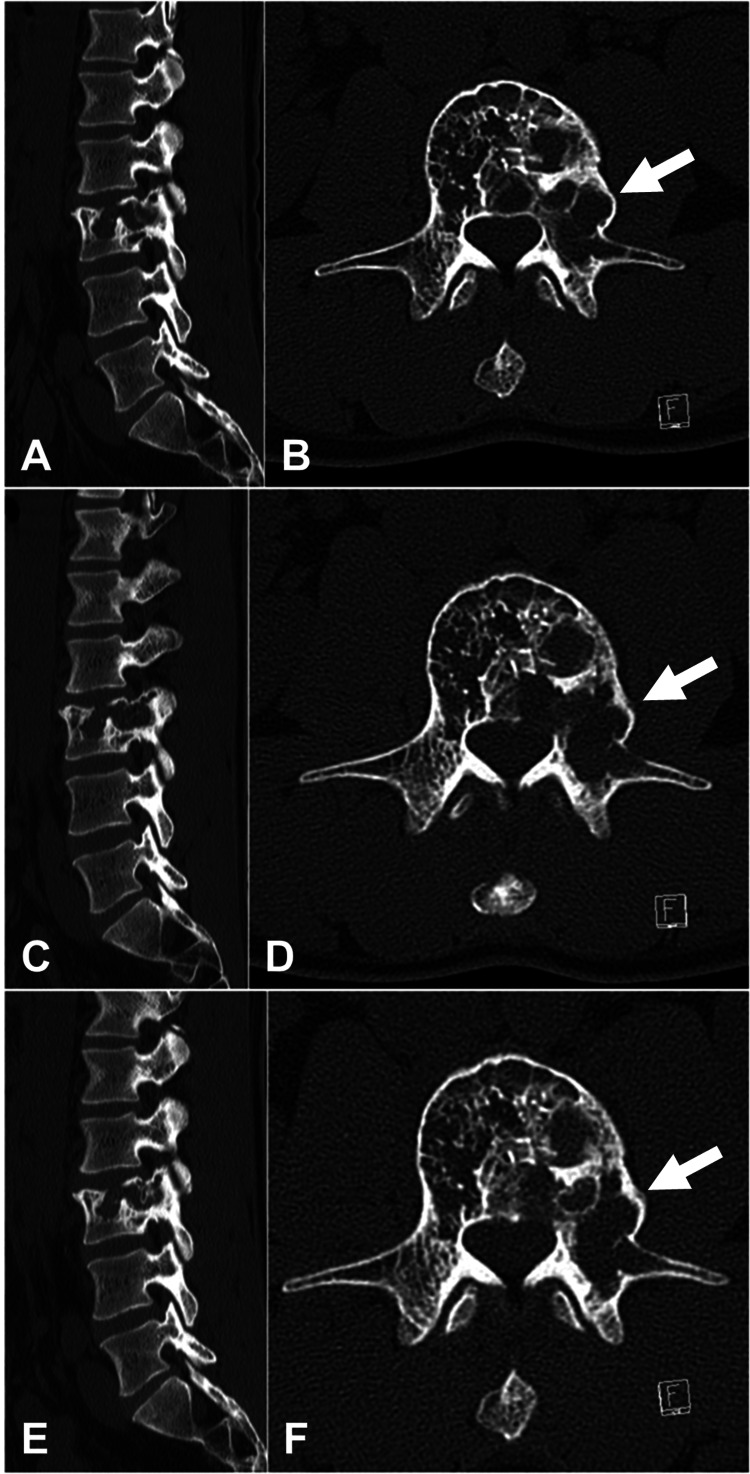
Reconstructed computed tomography. (A) At 16 weeks, decrease in the size and number of cysts, and most ossification of the lesion. (B) At 17 months, expansion of the erosive lesion. (C) After the re-treatment of denosumab had to be restarted, no evidence of recurrence on computed tomography. (A, C, and E) Sagittal view. (B, D, and F) Axial view.

No adverse events occurred during denosumab therapy. The pain in the lower back and left leg gradually improved, and at 16 weeks, the symptoms disappeared. The size and number of cysts were noted to decrease on CT images, and the lesion completely ossified over time (Figure 4A, 4B). Subsequently, he resumed his hobby of kickboxing. Once it was concluded that a satisfactory local effect was achieved, the therapy was stopped after three months. However, at follow-up after 17 months, despite being asymptomatic, the erosive lesion was expanded on CT images (Figure 4C, 4D). Hence, denosumab treatment was restarted at 60 mg subcutaneously once monthly. At the follow-up after 37 months, he had no pain in the lower back and leg, and there was no evidence of recurrence on CT images (Figure 4E, 4F).

## Discussion

Herein, we present a case of lumbar spinal ABC treated with denosumab. The lesion regressed with denosumab therapy, and no side effects were observed. Locally aggressive ABC lesions invade not only the vertebral body but also the posterior elements, such as the pedicle and lamina. Boriani et al. [10] reported 41 cases of ABCs of the spine, and the lesions involved the posterior elements and invaded the vertebral body in 26 cases (63%). Patients who underwent surgery for tumors of the posterior elements often developed postoperative complications. Hence, we often hesitate to perform surgeries in such cases. Boriani et al. [10] reported that complications after “en bloc” excision of spinal tumors were observed in 47 of 134 patients (35.1%), and three patients died because of such complications. In our case, it was difficult to remove the ABC because of the proximity to the spinal canal and foramina. In such cases, non-surgical treatment is often selected.

Zhu et al. [11] reported the long-term effectiveness of radiation therapy as an independent treatment option for eight patients with ABC. All patients were doing well at follow-up and had not experienced adverse reactions to radiation therapy. Amendola et al. [12] reported seven patients with ABC treated using embolization. Clinical and radiographic response was achieved in all patients who were completely free of disease, and none required surgery. In these reports, it was concluded that radiation therapy and embolization result in an excellent prognosis for patients with ABC. Percutaneous doxycycline was documented to have a favorable response in the treatment of ABCs. A recent study demonstrated clinical and imaging regression of disease with occlusion of the feeding artery with N-2-butyl-cyanoacrylate, which was then considered a novel therapy for the management of ABCs [13]. Also, Guarnieri et al. [14] reported percutaneous treatment of spinal ABCs with osteoconductive cement, and it is efficacious for the treatment of ABC.

Denosumab has recently been used to treat ABCs successfully [5,7]. Denosumab, a human monoclonal IgG2 antibody derived from mammalian cell lines, targets the receptor activator of NF-kB (RANK) ligand (RANKL), which is crucial for the differentiation of osteoclasts involved in bone resorption. Denosumab selectively binds to RANKL with high affinity, thereby preventing it from interacting with and activating the RANK receptor on the surface of osteoclasts and their precursors. Consequently, this inhibits the activation and differentiation of osteoclast-like giant cells and the resultant osteolytic damage. Dürr et al. [15] reported that six patients with ABCs were treated with denosumab, and two patients developed recurrence during the follow-up period. One patient had a sacral ABC that was treated with denosumab after treatment failure with embolization. The patient developed local recurrence one year after discontinuing denosumab, which was subsequently restarted. One and a half years later, radiological recalcification was confirmed. The second patient had an ABC of the pelvis and was administered denosumab after failure of curettage and bone grafting. The disease recurred one year later, curettage and bone grafting were repeated, and partial sclerosis was achieved after restarting denosumab. Kurucu et al. [7] described nine patients with ABCs treated with denosumab. All patients were asymptomatic after three months; however, four patients developed recurrence. Maximen et al. [16] reviewed the literature on the use of denosumab for ABC and reported that pain reduction and neurological improvement were maintained, with a recurrence rate of 18.6%. Based on these reports and our experience, recurrences of ABC may occur after denosumab termination, and the timing of treatment cessation is controversial. In some cases, recurrence of ABC occurred several years after the cessation of denosumab; therefore, long-term follow-up is necessary.

## Conclusions

We have reported the case of a 38-year-old Japanese male with lumbar ABC that involved the posterior portion of the vertebral body and left pedicle, resulting in radiculopathy. The optimal treatment of ABCs of the spine remains controversial; however, this case illustrates that single-therapy denosumab is an option for ABC.
